# Diastolic versus Systolic Left Ventricular Dysfunction as Independent Predictors for Unfavorable Postoperative Evolution in Patients with Aortic Regurgitation Undergoing Aortic Valve Replacement

**DOI:** 10.3390/medicina58111676

**Published:** 2022-11-19

**Authors:** Luminita Iliuta, Andreea Gabriella Andronesi, Camelia Cristina Diaconu, Horatiu Moldovan, Marius Rac-Albu, Madalina-Elena Rac-Albu

**Affiliations:** 1 Department of Medical Informatics and Biostatistics, University of Medicine and Pharmacy “Carol Davila”, 050474 Bucharest, Romania; 2 Cardioclass Clinic for Cardiovascular Disease, 031125 Bucharest, Romania; 3 Nephrology Department, University of Medicine and Pharmacy “Carol Davila”, 050474 Bucharest, Romania; 4 Nephrology Department, Fundeni Clinical Institute, 022328 Bucharest, Romania; 5 Internal Medicine Department, University of Medicine and Pharmacy “Carol Davila”, 050474 Bucharest, Romania; 6 Internal Medicine Clinic, Clinical Emergency Hospital of Bucharest, 014461 Bucharest, Romania; 7 Academy of Romanian Scientists, 3 Ilfov Street, 050044 Bucharest, Romania; 8 Department of Cardio-Thoracic Pathology, University of Medicine and Pharmacy “Carol Davila”, 050474 Bucharest, Romania; 9 Department of Cardiovascular Surgery, Clinical Emergency Hospital, 014461 Bucharest, Romania

**Keywords:** aortic valve replacement, aortic regurgitation, diastolic dysfunction, restrictive diastolic pattern, systolic dysfunction

## Abstract

*Background and Objectives*: Chronic severe aortic valve disease is associated with important changes in left ventricle (LV) performance associated with eccentric or concentric LV hypertrophy. We aimed to assess the immediate prognostic implications of the type of the LV diastolic filling pattern (LVDFP) compared with LV systolic performance in patients with severe aortic regurgitation (AR) undergoing aortic valve replacement (AVR) and to define the independent echographic predictors for the immediate and long-term prognoses. *Materials and Methods*: We performed a prospective study enrolling 332 AR patients undergoing AVR, divided into two groups: Group A—201 pts with normal LV systolic function, divided into two subgroups (A1: 129 pts with a nonrestrictive LVDFP and A2: 72 pts with restrictive LVDFP), and Group B—131 pts with LV systolic dysfunction (LV ejection fraction LVEF < 50%), divided into two subgroups (B1: 83 pts with a nonrestrictive LVDFP and B2: 48 pts with restrictive LVDFP). *Results*: The early postoperative mortality rate was higher in patients with a restrictive LVDFP (11.12% in A2 and 12.5% in B2) compared with normal LV filling (2.32% in A1 and 7.63% in B1, *p* < 0.0001), regardless of the LVEF. The restrictive LVDFP—defined by at least one of the following echographic parameters: an E/A > 2 with an E wave deceleration time (EDt) < 100 ms; an isovolumetric relaxation time (IVRT) < 60 ms; or an S/D ratio < 1 in the pulmonary vein flow—was an independent predictor for early postoperative mortality, increasing the relative risk by 8.2-fold. Other independent factors associated with early poor prognosis were an LV end-systolic diameter (LVESD) > 58 mm, an age > 75 years, and the presence of comorbidities (chronic obstructive pulmonary disease-COPD or diabetes mellitus). On a medium-term, an unfavorable evolution was associated with: an age > 75 years (RR = 8.1), an LV end-systolic volume (LVESV) > 95 cm^3^ (RR = 6.7), a restrictive LVDFP (RR = 9.8, *p* < 0.0002), and pulmonary hypertension (RR = 8.2). *Conclusions*: The presence of a restrictive LVDFP in patients with AR undergoing AVR is associated with both increased early and medium-term mortality rates. The LV diastolic function is a more reliable parameter for prognosis than LV systolic performance (RR 9.2 versus 2.1). Other independent predictors for increased early postoperative mortality rate were: an age > 75 years, an LVESD > 58 mm, and comorbidities (diabetes mellitus, COPD), and for unfavorable evolution at 2 years postoperatively: an age > 75 years, an LVESV > 95 cm^3^, and severe pulmonary hypertension.

## 1. Introduction

Chronic severe aortic valve disease is associated with important changes in LV performance associated with eccentric or concentric LV hypertrophy. Diastolic dysfunction has been demonstrated to precede the alteration in systolic function in these patients [1].

In patients with severe chronic aortic regurgitation (AR), chronic volume overload is suggested to be more harmful for the ventricle because of the increased end-diastolic and end-systolic volumes; thus, AR is more harmful than mitral regurgitation or aortic stenosis. On the other hand, LV systolic dysfunction is usually initially reversible with surgery, but diastolic dysfunction needs more time to ameliorate [2].

The diastolic dysfunction severity is closely related to the type and severity of the underlying valvular lesion. The presence of a restrictive LV diastolic filling pattern (LVDFP) determines a less-favorable prognosis. In addition, the change or lack of change in LV volume and systolic or diastolic performance within the first year after AVR seems to be an important predictor of long-term prognosis [3].

The medical literature evaluating the effects and efficacy of AVR in severe AR is composed of relatively small-population heterogeneous studies. The studies have a limited applicability in trying to formulate a unitary management of these patients, because they vary considerably in terms of the patient selection, surgical intervention, outcomes evaluated, and postoperative follow-up timing.

Some studies tried to establish the early, intermediate, and late consequences of AVR on LV size regression and the LVEF; whether the LV can return to normal dimensions; and how rapidly the myocardial hypertrophy dimensions and LV dysfunction regress after AVR [4,5,6].

In addition, the influence of the LVDFP on early postoperative evolution in patients with AR undergoing AVR has been evaluated in a few previous studies [7], but in the reviewed literature, there were no studies that compared the influence of systolic performance versus diastolic performance of the LV on the postoperative evolution in these patients.

However, short-term or long-term studies on the effects of AVR on the LV systolic and diastolic performance and the relation between LV dilatation reversal and the increase in LV systolic function have not been reported.

To address these issues, the first aim of our study was to investigate whether the diastolic or systolic performance of the LV predicts the outcome after AVR in patients with chronic severe AR. We aimed as well to assess the immediate and medium-term prognostic implications of the LVDFP in patients with AR undergoing AVR and to define the echographic parameters related to diastolic function that can be taken into consideration as independent predictors of the immediate and long-term evolution in these patients and their adjusted value for preoperative risk evaluation.

## 2. Materials and Methods

### 2.1. Subjects

We conducted a prospective study that enrolled 332 patients with AR undergoing AVR in the “C.C. Iliescu” Emergency Institute for Cardiovascular Diseases in three consecutive years. Informed consent was obtained from all subjects and the local ethical committee approved the study.

**Figure 1:**
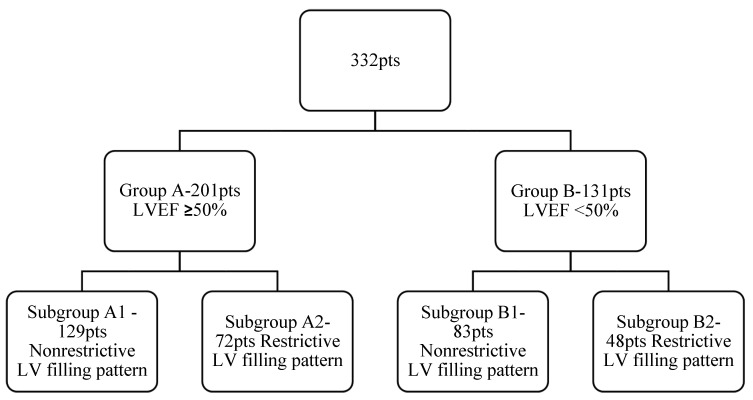
Study group structure depending on LV systolic and diastolic performance.

Selected patients were divided in two main groups, depending on their LV systolic dysfunction: Group A included 201 patients with normal LV systolic performance with an LVEF ≥ 50% and the second group, Group B, included 131 patients with LV systolic dysfunction with an LVEF < 50%. Taking into consideration the LV diastolic function, we subsequently divided each group into two: Group A was divided into Subgroup A1, with 129 patients with a nonrestrictive diastolic filling pattern (normal diastolic filling, mild or moderate diastolic dysfunction), and Subgroup A2, comprising 72 patients with a restrictive diastolic filling pattern (severe diastolic dysfunction). Similarly, patients from Group B were divided into Subgroup B1, comprising 83 patients with a nonrestrictive diastolic filling pattern, and Subgroup B2, comprising 48 patients with a restrictive diastolic pattern (Figure 1).

The exclusion criteria were as follows:

- Acute AR;
- Acute endocarditis;
- Associated severe aortic stenosis;
- Associated or previous mitral or tricuspid valve replacement or repair;
- Aortic dissection;
- Previous aortic valve surgery;
- Congenital diseases unrelated to AS;
- Coronary significant lesions (more than 50% coronary stenosis);
- Postoperative prosthesis mismatch;
- Permanent atrial fibrillation;
- Presence of a pacemaker;
- Left or right bundle-branch block.

Patients undergoing concomitant procedures such as associated ascending aortic surgery with AVR were not excluded. At the moment of enrollment, all patients were in sinus rhythm. Most of the patients were male (71%), the mean age of the whole group was 62 ± 16 years, and the mean LV ejection fraction (LVEF) was 52 ± 5%. Both groups were similar regarding age, sex, and comorbidities (see Table 1 for group characteristics).

All patients underwent AVR with a mechanical prosthesis (242 pts), AVR with a biological prosthesis (76 pts), or AVR combined with ascending aorta replacement (Bentall operation—12 pts, Wheat operation—2 pts).

The follow-up included both a clinical and echocardiographic evaluation (including TDI) before surgery and postoperatively at 10 days; 1, 3, and 6 months; and yearly for 4 years postoperatively. The clinical exams included the NYHA functional classification and quality-of-life scores.

### 2.2. Ultrasound Methods

We used a Philips Affinity30 or portable General Electric VIVID machine, with a 3.5 MHz probe for all echocardiographic examinations with techniques and calculations in accordance with the European and American Society of Echocardiography recommendations [8].

At each visit, the echographic parameters assessed included: the LV systolic and diastolic function (with complete TDI parameter measurements), the left atrium dimensions and indexed volume, and the LV end-systolic and end-diastolic dimensions (including the LV end-diastolic volume (LVEDV) and the LV end-systolic volume (LVESV)). Preoperatively, we evaluated the AR severity and postoperatively, we evaluated the parameters related with aortic prosthesis functionality.

The LV systolic performance was determined using a calculation of the LVEF using the volumetric Simpson method.

The assessment of diastolic function was based on a comprehensive echocardiographic study integrating all available two-dimensional and Doppler data [9,10]:

- Transmitral flow: measurement of transmitral flow parameters, including the early (E) and late (A) diastolic filling velocities, the E/A ratio, the E wave deceleration time (DT), and the isovolumetric relaxation time (IVRT) from an apical four-chamber view with a conventional pulsed-wave Doppler;
- Pulmonary venous flow: assessment of diastolic (D) and “forward” systolic (S) velocities into the left atrium, and the “backwards” late diastolic A reversal wave, which corresponded to the atrial contraction;
- Flow propagation velocity (Vp): measured using the color Doppler M mode from the apical four-chamber view with the M mode beam aligned parallel to the LV inflow and a calculation of the E/Vp ratio;
- Tissue Doppler imaging: assessment of LV relaxation rate by recording longitudinal velocities at the mitral annulus with the sample volume (2–5 mm) placed at the septal or lateral border of the mitral annulus in the apical four-chamber view for an estimation of the LV filling pressures using an average of the lateral and septal E/Ea ratio.

We considered restrictive diastolic filling to be present if either of the following echographic findings were found [11]:

- EDT < 150 ms, E/A ratio > 2, and E/Vp > 1.5;
- S/D < 1;
- IVRT < 60 ms with elevated filling pressures (E/Ea > 12);
- Ea/Aa < 1 [11].

Additionally, we preoperatively performed coronary arteriography in all patients over 35 years and for patients under 35 years old with angina pectoris. We did not include patients with associated significant coronary artery disease (more than 50% reduction in any coronary artery diameter). In addition, we used brain natriuretic peptide (BNP) titration for patients with a preserved LVEF and symptoms suggestive of heart failure (considering that diastolic heart failure was likely at a BNP > 100 pg/mL) [12].

The main endpoints tested were: NYHA class, LVDFP type, quality of life (appreciated on a scale from 1 to 10 using a questionnaire filled in by the patient at each visit), and death.

For the evaluation of life quality, we used a self-reported questionnaire with two parts: a mental score (MCS) and a physical one (PCS), both being continuous from ten to zero (with zero being the worst quality of life). We also asked the patients to report their perception of their quality of life during the follow-up at 30 days. The question asked, “How would you rate your quality of life now?” for both mental and physical aspects, with choices between “better than before your procedure,” “the same as before your procedure,” and “worse than before your procedure.”

The main null hypotheses tested were:

- LV systolic dysfunction and restrictive LVDFP are independent predictors for unfavorable postoperative evolution in patients with AR undergoing AVR;
- The presence of a restrictive LVDFP in preoperative risk evaluation in patients with AR undergoing AVR is more important than LV systolic dysfunction.

### 2.3. Statistical Analysis

All calculations were performed with the Statistical Package for the Social Sciences version 23 (SPSS 23) program. Numerical data were synthesized as a mean ± standard deviation, and qualitative data were recorded as percentages. Qualitative data were tested using the Pearson chi-squared test, likelihood ratio, and Fisher’s exact test and quantitative data were tested between the two groups with an independent samples *t*-test and for three groups with ANOVA. In order to compare the two groups, a univariate logistic regression analysis was used. In addition, the association between preoperative data and the magnitude of postoperative change in LV dimensions and function was tested using a linear regression analysis.

A multivariate logistic regression analysis was performed to identify predictive factors for mortality.

The area under the receiver operating characteristic (ROC) curve and the Hosmer–Lemeshow goodness-of-fit statistic test were calculated to assess the discrimination and calibration of the model, respectively. To evaluate the goodness of fit of the model, the Cox and Snell/Nagelkerke value was calculated. A value of *p* < 0.05 was considered statistically significant.

## 3. Results

We calculated the overall percentage of patients whose self-reported quality-of-life scores (SR QOL score) changed. A total of 39.8% of the patients from Group A had a preoperative SR QOL score less than 5, compared to 84.734% of the patients from Group B (*p* < 0.005, likelihood ratio). At 30 days postoperatively, 59.70% of the patients from Group A reported a better postoperative SR QOL score, compared with only 34.35% in Group B with preoperative LV systolic dysfunction (*p* < 0.005, likelihood ratio).

The postoperative echocardiography showed a trend towards an improvement in the LVEF in Group B (*p* = 0.06) and a significant improvement in Group A (*p* = 0.002). The postoperative end-diastolic and end-systolic dimensions decreased significantly in both groups (Table 2).

Following surgery, the early mortality rate (defined as death within 1 to 365 days after the date of the AVR surgery) was different in patients with a restrictive LVDFP compared to those with a nonrestrictive LVDFP. Thus, the early postoperative mortality rate was significantly higher in patients with an abnormal LVDFP (11.12% in Subgroup A2 and 12.5% in B2) compared with patients with normal LV filling (2.32% in A1 and 4.82% in B1), regardless of the LV systolic performance (*p* < 0.0001). After 1 year postoperatively, the mortality rate was also influenced by the LV systolic performance (5.47% in Group A vs. 7.63% in Group B, *p* = NS). The cumulative incidence of death at 1 year from cardiovascular causes was 2.98% in Group A and 4.58% in Group B. Extracardiac causes (cancer-related, comorbidities, or other causes) were associated with a cumulative 1-year mortality of 1.99% in Group A, and 1.58% in Group B. Patients that experienced an intraoperative death were not included in the database. Approximately 1/5 of the patients (20,18%) had severe LV systolic dysfunction (LVEF < 35%) and a severely enlarged LV with an LVESD > 58 mm, being at the borderline indication for surgery, which can explain the high mortality rate.

Early postoperatively, a multivariate logistic regression analysis revealed that the main independent predictors for the evolution of the patients with AR undergoing AVR were:

- A restrictive LVDFP (RR between 6.9 and 9.1, *p* value = 0.001);
- A severe LV systolic dysfunction with an LVEF of less than 35% (RR = 2.1, *p* value = 0.002);
- Severe pulmonary hypertension with a mean PAP > 50 mmHg (RR = 3.4, *p* value = 0.021);
- A patient age of more than 75 years (RR = 7.2, *p* value = 0.013);
- The presence of preoperative AR (RR = 6.9, *p* value = 0.031);
- Comorbidities (DM, COPD) (RR = 7.6, *p* value = 0.071);
- A severely dilated LV with an LVES diameter > 58 mm and LVES volume > 200 mL/m^2^ (RR = 6.2–6.4, *p* value = 0.067).

**Figure 2:**
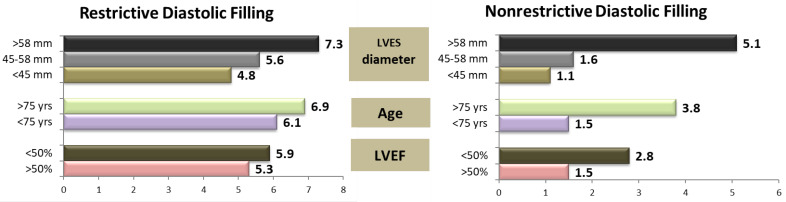
Early postoperative risk of death in patients with AVR depending on the type of LVDFP. LVEF—left ventricle ejection fraction; LVES—left ventricle end-systolic.

At 2 years postoperatively, the relative risk of death associated with the parameters known to increase the mortality rates in patients undergoing AVR (such as an increased LVES diameter, age, and LV systolic performance [13]) were higher in patients with severe LV diastolic dysfunction with a restrictive LVDFP (Figure 2).

In patients with mild or moderate diastolic dysfunction, severe LV dilatation (defined by an LVES diameter larger than 58 mm) and an age > 75 years markedly increased the risk of death (RR 5.1 and 3.8, respectively). Systolic dysfunction had a moderate influence on mortality, with an increase in the relative risk from 1.5 to 2.8. The presence of a restrictive LV diastolic filling pattern homogenized the relative risk values. In patients with this type of filling pattern, the postoperative risk of death significantly increased, regardless of the LV systolic performance (RR 5.3 versus 5.9), patient’s age (RR 6.1 versus 6.9), or presence of LV dilatation with an LVES diameter between 45 and 58 mm (RR 4.8 versus 5.6), proving an independent predictor value for severe diastolic dysfunction (*p* = 0.001). In these patients, the only independent predictor for increasing the risk of death was LV dilatation with an LVESD > 58 mm (RR 7.3) (Figure 1).

**Figure 3:**
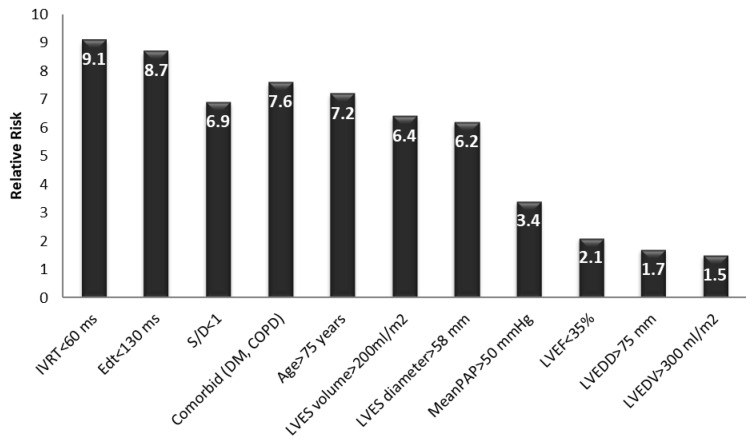
Early postoperative risk of death in patients with aortic regurgitation undergoing AVR. IVRT—isovolumetric relaxation time; DM—diabetes mellitus; COPD—chronic obstructive pulmonary disease; LVES—left ventricle end-systolic; PAP—pulmonary arterial pressure; LVEF—left ventricle ejection fraction; LVEDD—left ventricle end-diastolic diameter; LVEDV—left ventricle end-diastolic volume.

The presence of a restrictive LV diastolic filling pattern increased the risk of death early postoperatively by 8.2-fold, regardless of the presence of other parameters known to increase the mortality rate in patients with AR undergoing surgical treatment. Based on the regression analysis, the main independent echographic predictors for early postoperative death related to severe diastolic dysfunction in these patients were: an E wave deceleration time (EDt) < 130 msec with an E/A ratio > 2, an isovolumetric relaxation time (IVRT) < 60 ms, and a systolic per diastolic wave ratio < 1 (Figure 1) in the pulmonary artery vein flow. Additional independent risk factors included an age > 75 years, severe LV dilation (an LVES diameter > 58 mm or an LVES volume > 200 mL/m^2^), and the presence of comorbidities such as diabetes mellitus or COPD. The parameters of the LV systolic function, end-diastolic diameters, and volumes, as well as the presence of pulmonary hypertension, were not correlated with a significant increase in early postoperative mortality rate in these patients. Thus, the restrictive LVDFP turned out to be an independent predictive factor for an increasing early mortality rate in these patients (*p* = 0.001), regardless of the LV dimensions and systolic function, the patient’s age, comorbidities, or pulmonary hypertension (Figure 3).

**Figure 4:**
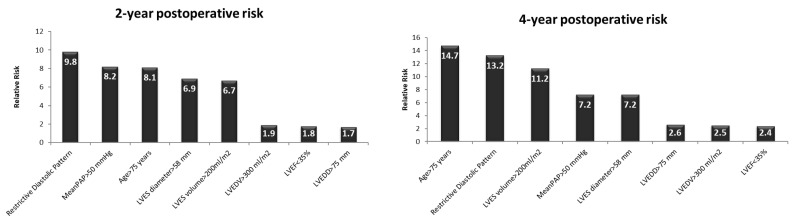
Risk of death at 2 and 4 years postoperatively in patients that received AVR for AR. LVES—left ventricle end-systolic; PAP—pulmonary arterial pressure; LVEF—left ventricle ejection fraction; LVEDD—left ventricle end-diastolic diameter; LVEDV—left ventricle end-diastolic volume.

On a medium term, at 2 and 4 years after the AVR, an unfavorable evolution was associated with an age > 75 years (RR = 8.1/2 yrs and 14.7/4 yrs, respectively, *p* < 0.01), severe LV dilatation (end-systolic volume > 200 mL/m^2^—RR = 6.7/2 yrs and 11.2/4 yrs, respectively, *p* < 0.0001 and LV end-systolic diameter > 58 mm—RR = 6.9/2 yrs and 7.2/4 yrs, respectively, *p* < 0.01), a restrictive LVDFP (RR = 9.8/2 yrs and 13.2/4 yrs, respectively, *p* < 0.0002), and severe pulmonary hypertension (RR = 8.2/2 yrs and 7.2/4 yrs, respectively, *p* < 0.01). The end-diastolic diameter, the volume of LV, and the LV systolic performance did not significantly influence the mortality rate at 2 and 4 years postoperatively in these patients (Figure 4).

**Figure 5:**
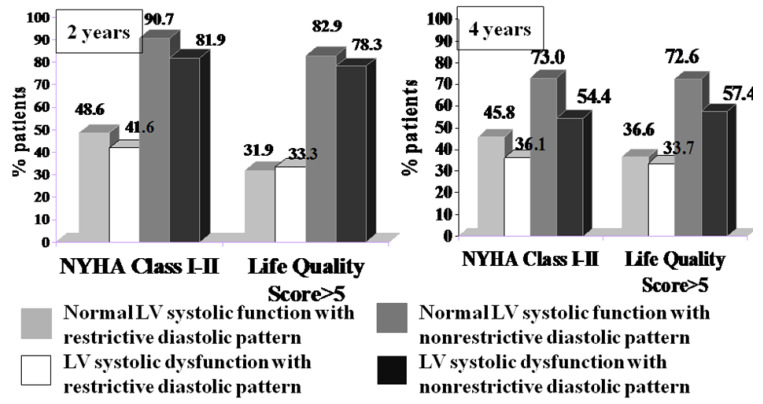
The percentage of patients with a favorable evolution depending on NYHA class and quality of life Score at 2 and 4 years postoperatively after AVR for AR. NYHA—New York Heart Association.

From a clinical point of view, patients with mild symptoms (NYHA class I or II and a life quality score > 5) were twice as likely to be in the group with nonrestrictive diastolic dysfunction, regardless of their LV systolic performance at both the 2- and 4-year follow-ups (Figure 5).

## 4. Discussion

Severe AR leads to an increase in both preload (volume overload) and afterload, which in turn causes a combination of chamber dilation and hypertrophy, usually eccentric, that is generally associated with the worsening of diastolic function. The LVEF is often initially within normal limits, and many patients with severe AR and subclinical myocardial dysfunction are asymptomatic or have only mild symptoms. However, when the compensatory mechanisms are exhausted, the changes in LV geometry and anatomy become irreversible, with negative consequences for survival and increased postoperative morbidity [14].

On the other hand, both the systolic and diastolic performance of LV have an important influence on the postoperative evolution of patients with AR undergoing AVR, which is why they should be investigated before the onset of irreversible LV dysfunction. In the majority of patients with chronic severe AR that receive AVR, usually within the first few months after surgery, there is a substantial reversal of LV dilatation that is associated with a significant increase in the LV systolic performance [15]. However, there are studies suggesting that patients who have preoperative irreversible LV dysfunction do not benefit from AVR. In these patients, valve replacement leads to less of a reduction in the LV dimensions and no or a small improvement in systolic performance, despite the correction of valvular regurgitation; unfortunately, the preoperative identification of these patients remains very difficult [7,16].

The echocardiographic evaluation of the diastolic performance in severe AR can be challenging. The regurgitation jet can interfere with the recording of mitral inflow velocities; therefore, the careful positioning of the sample volume is needed in order to avoid contamination with the AR jet. There are limited data on the accuracy of the estimation of LV filling pressures in chronic severe AR. This explains why, in these patients, it is important to take into account the presence of LA enlargement and the average E/Ea ratio in order to support the presence of increased LV filling. In patients with severe AR, whether acute or chronic, the premature closure of the mitral valve, diastolic MR, LA enlargement, and an average E/Ea ratio > 14 are consistent with elevated LV filling pressures. An invasive measurement of the LV filling pressure was well correlated with the echocardiographic findings [17].

After AVR, the LV systolic and diastolic performances are influenced by the LV contractility, the LV and left atrium dimensions and architecture, the LV afterload, and the extent of irreversible interstitial fibrosis. Thus, in order to prevent severe and irreversible LV function deterioration and to better predict postoperative outcomes, it is important to search for other parameters besides a low LVEF that are associated with a poor prognosis.

Because it is highly dependent on the left ventricular loading conditions, the primary relevance of the preoperative LVEF in patients with AR may not accurately reflect the intrinsic LV performance, and it is not a very good marker for the determination of optimal timing for surgical intervention and for postoperative course prediction.

On the other hand, the importance of diastolic dysfunction on the postoperative evolution in patients with AR is still underestimated. Severe diastolic dysfunction correlates well with other comorbidities known to increase mortality, such as diabetes mellitus, age, and secondary pulmonary hypertension [18,19]. The first two are known as causes per se of diastolic dysfunction, while pulmonary hypertension is usually associated with elevated left atrium pressure [20,21]. In addition, studies on patients with a transcatheter aortic valve implantation have shown that diastolic dysfunction is prevalent among these patients and is an independent predictor for poor intermediate-term survival, irrespective of comorbidities [22].

Moreover, patients with greater abnormalities in their LV filling pattern have a progressive increase in their risk of major cardiac events, and the stage of diastolic dysfunction correlates better than the LVEF with exercise impairment. In addition, in patients with heart failure, restrictive diastolic dysfunction is a stronger predictor of mortality than the LVEF [23,24,25]. These data are consistent with our findings, further supporting the importance of evaluating LV diastolic function.

In addition, the left ventricular function late postoperatively cannot be accurately predicted by routine measurements of the preoperative LVEF and LV dimensions, and a complete evaluation of the LVDFP should be more frequently used in clinical decision making and for recommending surgery in patients with severe AR.

In patients with severe AR, persistent diastolic dysfunction with a maintained LV systolic performance was observed late after successful AVR and can be explained by the incomplete regression of the extracellular matrix four years after the valve replacement [2]. In patients with severe aortic stenosis, after AVR, most studies have reported a regression of LV hypertrophy early postoperatively with an amelioration of LV diastolic performance [26].

In the majority of patients with chronic severe AR, AVR results in a substantial reversal of LV dilatation within the first few months after the operation, which is associated with a significant increase in LV systolic performance. Importantly, numerous studies have indicated that a depressed LV systolic performance may improve and, in some patients, normalize after the reversal of the volume overload by AVR [27]. These changes, which are observed during the first year after surgery, correlate with survival. Patients with a normal systolic function following surgery carry an excellent prognosis, whereas survival rates decrease significantly in patients with persistent LV dysfunction. However, in a subset of patients with preoperative left ventricular dysfunction (especially severe diastolic dysfunction, with systolic dysfunction having only a marginal effect), valve replacement leads to less of a reduction in the left ventricular diastolic volume or an increase in the LVEF, with a significant increase in both short-term and long-term mortality [28].

There is still a lot of uncertainty regarding whether to operate on a patient when the diastolic function is already severely depressed. There is no clear limit for a marked reduction in the LV diastolic function in surgical aortic disease, either by stenosis or regurgitation, and our knowledge is still limited regarding the outcome for patients with a restrictive LVDFP. The most recent guidelines underscore these limits when AVR is recommended in patients with a restrictive LVDFP [29].

The transition from a compliant (chronic compensated AR) to a rigid (decompensated AR) LV is due to the upregulation of fibroblast genes, with consequent remodeling of the extracellular matrix (ECM) of the heart [2]. The amount of thick collagen fibers is the highest in AR, and is significantly higher compared to that found in AS. The severity of heart remodeling correlates with impaired diastolic function and LV wall stress, but is not related to systolic dysfunction [30]. Irreversible fibrosis is accompanied by an alteration in the elastic properties, which leads to severe diastolic impairment with a restrictive filling pattern and is associated with a lack of reduction in LV size and a slow decrease in the LVEF, both in the short and long term postoperatively [3,31]. These mechanisms might explain why severe diastolic dysfunction is a better and more sensitive predictor of mortality than the LVEF in AR, and how the EF alone might be ineffective at choosing the best moment for surgical valve replacement.

We hypothesized that the operative and postoperative risk would be smaller in patients with milder LV diastolic dysfunction compared to patients with severe AR with a restrictive LVDFP. We also hypothesized that the rates of cardiovascular morbidity and mortality after surgery would not be so high and that AVR would improve the clinical outcome of most of these patients.

In general, the recognition of diastolic heart failure in the postoperative heart and its treatment remains difficult and often unsatisfactory. This condition often occurs in conjunction with some degree of systolic dysfunction. As the conditions under which diastolic dysfunction occurs vary between patients, straightforward therapeutic algorithms are not easy to provide for an individual. Although there are many studies on diastolic dysfunction, there are very few data about diastolic dysfunction in the postoperative cardiac surgical patient.

Although diastolic dysfunction is an important cause of heart failure, it remains underreported in the postoperative heart. We tried to find the criteria that could be applied to help diagnose it in this group of patients in the intensive care setting where cardiac surgical patients are usually managed in the immediate postoperative period. Since both systolic and diastolic heart failure share a similar clinical picture, it is important to recognize the difference between these two entities. Unfortunately, there is still very little evidence on how to treat diastolic dysfunction.

The clinical benefit for patients with mild diastolic dysfunction after AVR is significantly higher than for patients with advanced diastolic dysfunction. The deceleration time and E/A ratio are independent predictors of postoperative improvement in the LV systolic and diastolic performance and the LV dimensions in patients with severe AR and LV systolic dysfunction. The diastolic function has an increased sensitivity for defining a patient’s prognosis due to the alterations in the compliance and stiffness of the LV caused by irreversible remodeling. The complete assessment of diastolic function with tissue Doppler echocardiography is a noninvasive, simple, and reliable preoperative method that can be used to predict the short- and long-term outcomes after AVR and may help in the management of postoperative treatment in patients with severe AR undergoing AVR.

### Limitations

In our study, for the echographic evaluations, we did not use three-dimensional speckle-tracking, which is known as a very good technique for assessing the LV systolic function using echocardiography. This was because of technical limitations (only one ultrasound device available at this facility) and also because the required examination time was dependent on a good acoustic window, quality data sets, and on patient cooperation for breath-holding, limiting its feasibility in postoperative patients.

In addition, for the assessment of diastolic performance, only a small number of patients underwent an invasive determination of the LV filling pressures. However, the echocardiographic examinations included all the parameters indicating increased LV-filling pressures in patients with valvular heat disease, taking into account their limitations. The restrictive pattern was considered only when three parameters were modified. For the same reason, we didn’t include patients with atrial fibrillation in our study.

All the patients in our study benefited from surgical AVR without having a comparison with a possible control group in which transcatheter implantation was performed. For the data analysis, we did not take into account the duration of the surgery and intraoperative complications, but all operations were scheduled, not urgent. In addition, we did not analyze the causes of in-hospital deaths, but most of them occurred in the ICU by severe LV dysfunction or multiple systemic organic failure.

In our study, we found that the global LVEF had only a borderline predictive value in multivariable models that included restrictive diastolic filling pattern. This may be due to the fact that systolic function was preserved in many patients and because the prognostic data obtained from depressed systolic function may be obtained from other covariates that were included in the multivariate model. In addition, most of our patients with low EF had only mild or moderate systolic dysfunction (mean EF = 50% ± 2).

We recognize that the quality of the data regarding the causes of death is dependent on the quality with which the physicians certified the cause of death. Additionally, although the data quality in this study was high and the follow-up was complete, the cause-specific death rates in the study cohort were not established. Furthermore, although we did not examine mortality and causes of death, we examined other aspects of health following AVR, such as quality of life and the HF evolution. A lower early survival after AVR was explained by an increased relative risk of cardiovascular death, probably because of a too-late surgical indication and operating on patients with terminal heart failure. Future studies should focus on the role of earlier surgery in patients with asymptomatic aortic regurgitation and on optimizing the treatment and follow-up after AVR.

## 5. Conclusions

The presence of a restrictive LVDFP in patients undergoing AVR for AR was associated with an increased mortality rate early after surgery. Compared to LV systolic function, the LV diastolic function was a more reliable prognostic parameter.

The independent predictors for an increased early postoperative mortality rate early after surgery were: an age > 75 years, comorbidities (diabetes mellitus, COPD), a restrictive diastolic filling pattern (an EDt < 130 ms, an IVRT < 60 ms, or an S/D ratio < 1), an LV end-systolic diameter > 58 mm, and an LV end-systolic volume > 200 mL/m^2^.

The major independent predictors for an unfavorable evolution at 2 and 4 years after surgery were: an age > 75 years, severe pulmonary hypertension, a restrictive LVDFP, an LV end-systolic diameter > 58 mm, and an LV end-systolic volume > 200 mL/m^2^.

## Figures and Tables

**Table 1:** Baseline characteristics of study patients (n = 332).

	Group A—201 pts LVEF > 50%	Group B—131 pts LVEF < 50%
Mean (±SD) age (years)	62 (±16)	63 (±14)
Age > 75 years	20 (9.95%)	14 (10.69%)
% Female	60 (29.85%)	37 (28.24%)
Mean (SD) weight (kg)	75 (±15)	77(±14)
Mean (SD) height (cm)	172 (±9)	171 (±10)
Mean (SD) heart rate/24 h	78 (±15)	79 (±14)
Mean PAP > 50 mmHg	65 (32.34%)	50 (38.17%)
Restrictive diastolic pattern	72 (35.82%)	48 (36.64%)
Mean (SD) systolic blood pressure (mmHg)	148 (±22)	143 (±23)
Previous episodes of atrial fibrillation	25 (12.44%)	26 (19.85%)
Hypertension	125 (62.19%)	75 (57.25%)
Diabetes mellitus	51 (25.37%)	34 (25.95%)
COPD	45 (22.39%)	36 (27.48%)
Renal failure	20 (9.95%)	15 (11.45%)
NYHA class I/II	104 (51.74%)	7 (5.34%)
NYHA class III	60 (29.85%)	59 (45.04%)
NYHA class IV	37 (18.41%)	65 (49.62%)

SD—standard deviation; COPD—chronic obstructive pulmonary disease (>= Gold II); NYHA—New York Heart Association; PAP—pulmonary arterial pressure.

**Table 2:** Comparison of preoperative and postoperative echocardiographic variables.

Echographic Variables	Group A—201 pts LVEF ≥ 50%				Group B—131 pts LVEF < 50%			
	Before Surgery	6 Months after Surgery	2 Years after Surgery	*p* ^1^	Before Surgery	6 Months after Surgery	2 Years after Surgery	*p* ^1^
LV end-diastolic dimension (mm)	63 ± 6	58 ± 9	55 ± 8	0.081	65 ± 6	62 ± 8	59 ± 7	0.012
LV lateral wall thickness (mm)	14 ± 0.2	13 ± 0.2	12 ± 0.2	0.045	13 ± 0.2	12 ± 0.4	11 ± 0.2	<0.001
IVS thickness (mm)	13.0 ± 0.4	12.4 ± 0.5	11.7 ± 0.7	0.051	14.0 ± 0.4	13.0 ± 0.2	11.4 ± 0.4	<0.001
Mean (SD) LVEF (%)	59 (5)	62 (12)	63(10)	0.002	36 (4)	38 (5)	42 (5)	0.066
EDt (ms)	179.95 ± 60	184.72 ± 65	230.35 ± 74	0.051	162 ± 6	171 ± 8	177 ± 7	0.061
IVRT (ms)	119.5 ± 74	120 ± 44	123 ± 48	0.042	57 ± 2	61 ± 4	63 ± 2	0.034
E/A	1.6 ± 0.4	1.54 ± 0.5	1.47 ± 0.7	0.071	2.1 ± 0.3	2.0 ± 0.2	1.8 ± 0.4	0.043
Ea/Aa	1.2 ± 0.2	1.31 ± 0.3	1.38 ± 0.2	0.084	1.1 ± 0.3	1.2 ± 0.3	1.3 ± 0.4	0.079
E/Ea	10.2 ± 0.6	10.1 ± 0.3	9.5 ± 0.5	0.027	12.5 ± 0.6	12.7 ± 0.5	11.7 ± 0.6	0.043

LV—left ventricle; IVS—interventricular septum thickness; LVEF—left ventricle ejection fraction; IVRT—isovolumetric relaxation time; Ea—early diastolic velocity; Aa—late diastolic velocity. ^1^ ANOVA test.

## Data Availability

All data generated or analyzed during this study are included in this published article.
